# Supplementary material for: Effects of Soluble Dextrin Fiber from Potato Starch on Body Weight and Associated Gut Dysbiosis Are Evident in Western Diet-Fed Mice but Not in Overweight/Obese Children
Source: Nutrients. 2024 Mar 22;16(7):917. doi: 10.3390/nu16070917 (PMC11013109; doi:10.3390/nu16070917)
Supplement: Supplementary file 1 [file nutrients-16-00917-s001.zip › Supplementary Table S5.pdf]

Supplementary Table S5. Effects of soluble dextrin fiber (SDexF) treatment for 28 weeks on the relative fecal abundances (ng/100 mg of stool) of short-chain fatty acids (SCFAs) and amino acids (AAs) at the end of the experiment in female and male mice fed a normal diet (ND) and Western diet (WD).

Metabolite	Females, normal diet					Males, normal diet				
			SDexF		P value			SDexF		P value
	Mean	SD	Mean	SD		Mean	SD	Mean	SD	
Formic acid	30368	18741	19381	4738	0,0617	20593	5036	28361	15307	0,1091
Acetic acid	181467	36339	206735	66078	0,2582	164656	33772	220472	82853	0,0418
Propanoic acid	74653	15894	72377	24977	0,7925	67384	14682	65919	19964	0,8395
Isobutyric acid	10103	4437	7410	1825	0,0648	8232	1152	9551	2995	0,1687
Butyric acid	61494	19827	72602	29033	0,2856	57488	40076	75880	26335	0,1976
Pentanoic acid	15465	6047	10861	2853	0,0261	11772	2984	13683	5310	0,2889
Hexanoic acid	7749	4604	4986	1308	0,0580	5271	1306	7188	3983	0,1274
Alanine	69830	18913	30498	12660	0,0000	68912	33015	89928	52493	0,2529
Glycine	45363	16416	24781	6486	0,0002	37632	12048	58023	36170	0,0774
Valine	53214	18140	28867	8654	0,0002	48033	17878	82904	59908	0,0663
Leucine	61279	22515	32747	9194	0,0002	54629	20692	93993	68170	0,0687
Isoleucine	47560	15346	25689	7752	0,0001	44798	18301	76681	56547	0,0766
Proline	40007	15418	22071	5365	0,0004	32747	9208	44355	28424	0,1921
Methionine	79953	38756	44510	11828	0,0033	61388	17419	78340	38830	0,1815
Phenylalanine	63796	26982	36654	8991	0,0016	52460	16653	79924	48145	0,0752
Glutamic Acid	155193	51693	68423	45992	0,0001	126586	68657	161452	98587	0,3257
Tyrosine	92096	49159	52920	14193	0,0087	67989	18706	93233	50463	0,1184
	Femalers, Western Diet					Males, Western Diet				
			SDexF		P value			SDexF		P value
	Mean	SD	Mean	SD		Mean	SD	Mean	SD	
Formic acid	35146	14027	36842	13742	0,7649	31789	9340	33821	17067	0,7124
Acetic acid	123603	50328	148277	64707	0,2940	109383	35490	144673	49234	0,0500
Propanoic acid	40696	11772	53356	17606	0,0421	36100	10106	44201	6875	0,0294
Isobutyric acid	11981	4280	11916	3082	0,9666	10457	1888	12469	3012	0,0553

Butyric acid	29815	10049	27637	10777	0,6073	24117	5047	38067	12352	0,0010
Pentanoic acid	16015	6086	15701	5596	0,8956	13854	3426	15911	6345	0,3184
Hexanoic acid	9045	3736	9439	3745	0,7956	7949	2305	8595	4563	0,6552
Alanine	77087	21300	51829	8206	0,0012	66984	19600	85036	25338	0,0572
Glycine	54082	12420	46905	13765	0,1846	50378	13114	59556	12890	0,0913
Valine	81775	20566	59455	13094	0,0047	76098	23341	88143	19726	0,1786
Leucine	88307	22761	64864	16440	0,0086	82494	23328	97203	21014	0,1123
Isoleucine	75780	20384	52105	11463	0,0022	71249	21332	81074	20980	0,2581
Proline	41948	12038	40519	13602	0,7832	37312	10147	43007	13572	0,2444
Methionine	92962	31019	88420	32994	0,7269	82443	20277	91685	35412	0,4268
Phenylalanine	73995	20149	66639	21363	0,3866	67616	16773	76962	20274	0,2203
Glutamic Acid	183611	94049	90660	23556	0,0041	143751	63130	139329	56490	0,8557
Tyrosine	105153	37890	104288	39710	0,9563	93477	25015	104011	43431	0,4604

soluble dextrin fiber (SDexF)
